# Peritoneal dialysis discontinuation: to the root of the problem

**DOI:** 10.1007/s40620-023-01759-w

**Published:** 2023-09-25

**Authors:** Paola Piarulli, Valerio Vizzardi, Federico Alberici, Hilary Riva, Marta Aramini, Luca Regusci, Pietro Cippà, Antonio Bellasi

**Affiliations:** 1 Division of Nephrology and Dialysis, Department of Medical and Surgical Specialties, Radiological Sciences, and Public Health, University of Brescia and ASST Spedali Civili, Brescia, Italy; 2 Servizio di Nefrologia, Ospedale Regionale di Lugano, Ospdeale Civico, Ente Ospedaliero Cantonale, Via Tesserete 46, 6903 Lugano, Switzerland; 3 Servizio di Nefrologia, Ospedale Regionale di Mendrisio, Ente Ospedaliero Cantonale, Mendrisio, Switzerland; 4 Servizio di Chirurgia, Ospedale Regionale di Mendrisio, Ente Ospedaliero Cantonale, Mendrisio, Switzerland; 5 Università della Svizzera Italiana (USI), Lugano, Switzerland

**Keywords:** Peritoneal dialysis, Hemodialysis, Discontinuation, Survival, Outcome

## Abstract

As the global burden of chronic kidney disease continues to increase, the use of peritoneal dialysis is often advocated as the preferred initial dialysis modality. Observational studies suggest a survival advantage for peritoneal dialysis over hemodialysis for the initial 2–3 years of dialysis. Peritoneal dialysis has been associated with better graft survival after kidney transplantation and has a reduced cost burden compared to hemodialysis. However, several medical and non-medical reasons may limit access to peritoneal dialysis, and less than 20% of patients with end-stage kidney disease are treated with peritoneal dialysis worldwide. In this narrative review, we sought to summarize the recent medical literature on risk factors for peritoneal dialysis discontinuation, distinguishing the early and the late phase after peritoneal dialysis initiation. Although the definition of clinically relevant outcomes varies among studies, we observed that center size, older age, and the presence of many comorbidities are risk factors associated with peritoneal dialysis discontinuation, regardless of the phase after peritoneal dialysis initiation. On the contrary, poor technique training and late referral to nephrology care, as opposed to the need for a caregiver, patient burnout and frequent hospitalizations, are related to early and late peritoneal dialysis drop-out, respectively. The aim of the review is to provide an overview of the most relevant parameters to be considered when advising patients in the selection of the most appropriate dialysis modality and in the clinical management of peritoneal dialysis patients. In addition, we wish to provide the readers with a critical appraisal of current literature and a call for a consensus on the definition of clinically relevant outcomes in peritoneal dialysis to better address patients' needs.

**Graphical abstract:**

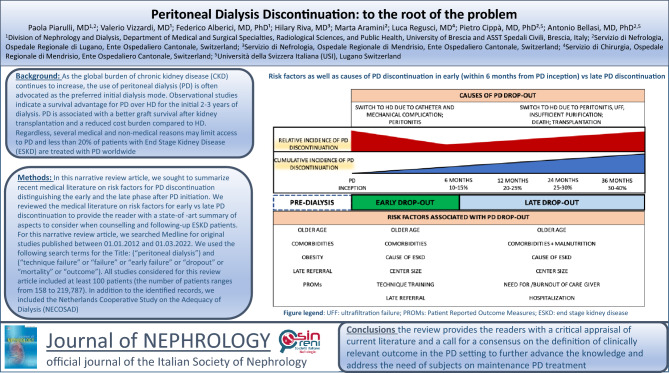

## Introduction

With the increasing global burden on public health-related chronic kidney disease worldwide, optimization of renal replacement therapy becomes a priority [1]. Several studies have compared hemodialysis (HD) and peritoneal dialysis (PD) [2] and, although evidence is far from being conclusive, the two modalities are generally considered equivalent overall [3]. Large observational studies indicate a survival advantage of PD for the initial 2–3 years of therapy, with subsequent loss of this benefit or later advantage to HD. Moreover, PD may offer some advantages over HD with regard to quality of life and kidney transplantation outcome [4, 5] and costs savings [1]. Ideally, the individual choice for the most appropriate dialysis modality should be made following an evidence-based approach considering patient characteristics and needs, current and future options for renal replacement therapy (RRT), and patient preferences. However, given the uncertainty regarding the impact of dialysis modality on hard outcomes, it often relies on local expertise, and financial incentives [1]. As a result, PD is under-prescribed compared to HD in many countries and only about 10–20% of patients with end-stage kidney disease (ESKD) worldwide undergo this treatment [6, 7], with a significant increase in the use reported in various countries such as China, Hong Kong, Thailand and USA due to adapted reimbursement and health care policies in the last years [1].

The first step for an evidence-based approach to support the choice of dialysis modality relies on the identification of factors associated with a specific outcome of interest. The focus of this article is on risk factors for PD discontinuation. Our aim is to summarize the data from recent literature on PD discontinuation and dropout to help physicians choose which patients could benefit from PD, avoiding PD under- and over-prescription, identifying at-risk patients to prioritize support resources in order to keep them on PD, and reducing the distress burden related to a switch of dialysis modality (hospitalization, need for vascular access creation for hemodialysis, etc.). Several studies have been conducted to identify factors that predict PD discontinuation in different populations or geographical areas as well as time periods, yielding different and sometimes conflicting results. In addition, not all studies distinguished among factors associated with early (within 6 months after PD initiation) vs late (afterwards) PD discontinuation, a distinction of major clinical relevance. We reviewed the medical literature on risk factors for early vs late PD discontinuation to provide the reader with a state-of-the-art summary of aspects to consider when advising and following-up ESKD patients. For this narrative review article, we searched Medline for original studies published between 01.01.2012 and 01.03.2022. We used the following search terms for the Title: (“peritoneal dialysis”) and (“technique failure” or “failure” or “early failure” or “dropout” or “mortality” or “outcome”). All studies considered for this review included at least 100 patients (the number of patients ranges from 158 to 219,787). In addition to the identified records, we included the Netherlands Cooperative Study on the Adequacy of Dialysis (NECOSAD) [8] study because this is the first study which used a time-dependent approach to define early vs late PD discontinuation and to analyze PD technique survival and all-cause mortality separately.

## Early vs late PD discontinuation definition

The overall incidence of PD discontinuation (defined as shift to hemodialysis), greatly varies among studies, ranging between 6 and 40% [9–11]. In general, PD discontinuation has been associated with older age at PD start, presence of diabetes mellitus and recurrent episodes of peritonitis [8–15]. However, the large heterogeneity in clinical and laboratory data included, as well as the lack of consistency in the definition of “discontinuation” among studies complicates the interpretation and the generalizability of these findings (Table 1). Indeed, some studies define PD discontinuation as the shift to HD irrespective of the duration and the cause of PD interruption [8, 11, 16]. Other studies define it as a shift to HD for more than 30 days [10, 12, 13, 17] or 2 months [9, 15] Alternatively, PD discontinuation has been defined as the occurrence of death by any cause [8, 11, 12, 16]. Similarly, censoring for death or renal transplantation has been carried out inconsistently across studies and, although PD vintage is associated with discontinuation, there is no consensus on the definition of early *vs* late PD discontinuation (Table 1).

**Table 1:** Definitions of Peritoneal Dialysis discontinuation according to different studies

Study	Definition and timing of PD discontinuation
Prognostic value of predialysis indices for technique failure and mortality in peritoneal dialysis patients (2017) Ther Apher Dial. 21(5):493–499 [15]	Combined use of HD and transition to HD for more than 2 months and all‐cause mortality within 2 years after PD commencement
Prognostic implications of predialysis patients’ symptoms in peritoneal dialysis patients (2021) Renal Failure. 43(1):216–222 [24]	All cause-mortality on PD
Time-dependent reasons for peritoneal dialysis technique failure and mortality (2010) Perit Dial Int. 30:170–177 [8]	Permanent switch to HD or death on PD
Early failure in patients starting peritoneal dialysis: a competing risks approach (2014) Nephrol Dial Transplant. 29: 2127–2135 [9]	Early PD discontinuation: transfer to HD during the first 6 months on PD and lasting more than 2 months (death and renal transplantation during the first 6 months on PD were considered as competing events)
Risk predictors and causes of technique failure within the first year of peritoneal dialysis: an Australia and New Zealand Dialysis and Transplant Registry (ANZDATA) Study (2018) Am J Kidney Dis. 72(2):188–197 [13]	Technique discontinuation within the first year, defined as transfer to HD for ≥ 30 days, or death
Predictors of outcomes in patients on peritoneal dialysis: a 2-year nationwide cohort study (2019) Sci. Rep. 9:3967 [14]	All-cause mortality on PD
Early technique failure in peritoneal dialysis patients in a multi‐ethnic Asian country (2020) International Urology and Nephrology. 52:1987–1994 [17]	Discontinuation within the first year, defined as transfer to HD for ≥ 30 days, or death
Peritoneal dialysis and mortality, kidney transplant, and transition to hemodialysis: trends from 1996–2015 in the United States (2020) Kidney Med. 2(5):610–619 [12]	Transfer to in-center HD, or death
Predictors of technique failure and mortality on peritoneal dialysis: an analysis of New Zealand peritoneal dialysis registry data (2021) :530–540. [24]	Transfer to HD for ≥ 90 days (the secondary outcomes were time to all-cause mortality)
Analysis of risk factors and construction of prediction model of drop out from peritoneal dialysis (2021) Medicine. 100:3(e24195) [11]	Death, transfer to hemodialysis, and kidney transplantation
Treatment practices and outcomes in incident peritoneal dialysis patients: the Swedish Renal Registry 2006–2015 (2021) Clinical Kidney Journal. 14(12):2539 2547 [16]	Transfer to HD, PD-related peritonitis and kidney transplantation
Peritoneal dialysis modality failure in a middle-income country: a retrospective cohort study (2021) Kidney Med. 3(3):335–342 [10]	Transfer to HD for ≥ 30 days
Technique failure in peritoneal dialysis: Modifiable causes and patient-specific risk factors (2022) Perit Dial Int. https://doi.org/10.1177/08968608221077461 [26]	Transfer to in-center HD for ≥ 30 days, or death
Importance of non-medical reasons for dropout in patients on peritoneal dialysis (2020) Clinical and Experimental Nephrology. 24:1050–1057 [27]	Transfer to HD or death

Some effort has been made to determine and validate an evidence-based definition of PD technique survival and to create a framework for reporting research. Lan and coworkers [18] investigated the influence of time spent on HD to define PD discontinuation and the likelihood of resuming PD after PD discontinuation (i.e., discontinuation is defined depending on the definition being analyzed if a patient remains on HD more than 30, 60, 90, 180, 365 days, respectively). They used data concerning 16,612 incident PD patients receiving care in Australia and New Zealand, and for these analyses transplantation, deaths occurring after transplantation, loss to follow-up, end of follow-up (31 December, 2012) and/or recovery of native kidney function were not considered a discontinuation and were censored. Although the overall technique survival rates were comparable among the different time definitions of PD discontinuation, the causes of PD discontinuation as well as the probability of resuming PD after discontinuation was significantly lower for longer periods of time spent on HD (the likelihood of returning to PD was 24%, 3%, 0.8% after 30, 180 and 365 days spent on HD, respectively).

**Fig. 1:**
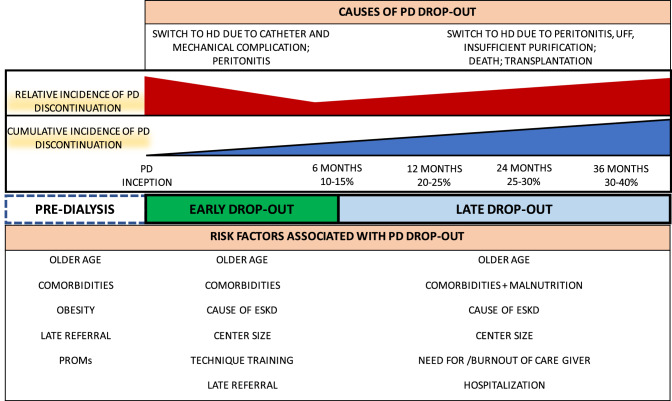
Risk factors as well as causes of PD discontinuation in early (within 6 months from PD inception) vs late PD discontinuation. *UFF* ultrafiltration failure, *PROMs* patient reported outcome measures, *ESKD* end stage kidney disease

In another observational study by Guo and Mujais [19], time on PD was associated with the risk of discontinuation. In this study, the rate of transfer to HD was highest during the first 6 months since PD start. Indeed, this is a period of increased patient vulnerability which requires special attention and care since it is associated with a higher risk of complications. Although not universally accepted, other studies used this time frame to define “early vs late PD discontinuation” [9, 20] (Fig. 1).

Consistently, we herein provide an overview of factors associated with PD discontinuation defined as pre-dialysis, early discontinuation and factors leading to PD discontinuation independently of the time spent on dialysis and/or after the first year from PD start.

### Pre-dialysis factors associated with PD discontinuation

Only a few studies attempted to identify pre-dialysis factors linked to PD discontinuation (Table 2). A Japanese study [15] reviewed demographic, clinical and laboratory data collected during the three months before dialysis inception of 158 subjects incident to PD between the years 1997 and 2013. In particular, they tested whether age, sex, body mass index (BMI), presence of hypertension, diabetes, dyslipidemia, smoking, laboratory parameters, current medication, and the Charlson Comorbidity Index (CCI) were associated with the risk of switching to HD (for longer than 2 months) and all-cause mortality during the first 2 years of PD. In the multi‐variable adjusted model, the authors observed that older age, higher levels of albumin, overweight/obesity, and hypocalcemia before PD start were independently associated with PD discontinuation and mortality. They also noticed that the ability of one single biomarker to identify patients who experienced PD discontinuation and mortality was much lower than when a combination of factors was used.

**Table 2:** Risk factors, causes and rate of PD discontinuation

Study	Factors associated with increased risk of PD discontinuation	Main causes of discontinuation	Discontinuation rate
*Pre-dialysis*			
Prognostic value of predialysis indices for technique failure and mortality in peritoneal dialysis patients (2017) Ther Apher Dial. 21(5):493–499 https://doi.org/10.1111/1744-9987.12546 [15]	Older age (≥ 65 years) Albumin (≤ 3.5 g/dL) Overweight/obesity Hypocalcemia before PD start (≤ 8.4 mg/dL)	Heart failure (41%) Peritonitis (22%) Inadequate dialysis dose (10%) Malignancy requiring surgery (10%)	17% (27/158) transfer to HD^e^ 8.9% (14/158) combination use of HD^e^ 5.7% (9/158) death from any cause^e^
Prognostic implications of predialysis patients’ symptoms in peritoneal dialysis patients (2021) Renal Failure. 43(1):216–222 https://doi.org/10.1080/0886022X.2021.1871920 [23]	Nausea (all-cause mortality) Anorexia (long-term mortality)	–	3.1% (28/898) transfer to HD 42.8% (384/898) death
*Early discontinuation*			
Adapted from: Time-dependent reasons for peritoneal dialysis technique failure and mortality (2010) Perit Dial Int. 30:170–177 https://doi.org/10.3747/pdi.2008.00277 [8]	Loss of residual renal function^a^ Older age^a^ Diabetes, CV disease^a^ Female^a^	Death (about 25%)^a^ Psychosocial/unknown (about 20%)^a^ Catheter complications (15%)^a^ Transplantation (13%)^a^ Infections (about 10%)^a^ Under dialysis/UFF/abdominal complications (about 17%)^a^	13% (during the first year)
Early failure in patients starting peritoneal dialysis: a competing risks approach (2014) Nephrol Dial Transplant. 29:2127–2135 https://doi.org/10.1093/ndt/gft055 [9]	HD before PD Transplant failure Early peritonitis Center size (< 20 new patients/year)	Catheter dysfunction (18.1%) Psychosocial reasons (16.4%) Miscellaneous reasons related to PD (15.5%) Peritonitis (15.3%) Miscellaneous reasons unrelated to PD (13.3%) Dialysis inadequacy (11.9%) Ultrafiltration failure (7.8%) Malnutrition (1.8%)	6.3% (615/9675) transfer to HD^b^ [of note, competing events: 2.2% (215/9675) kidney transplantation^b^; 8.4% (809/9675) death^b^]
Risk predictors and causes of technique failure within the first year of peritoneal dialysis: an Australia and New Zealand Dialysis and Transplant Registry (ANZDATA) Study (2018) Am J Kidney Dis. 72(2):188–197 https://doi.org/10.1053/j.ajkd.2017.10.019 [13]	Older age (> 70 years) BMI < 18.5 kg/m^2^ Diabetes History of ischemic heart disease, cerebrovascular disease or peripheral vascular disease Prior RRT Late referral to nephrology care Smaller centers	Death (32.2%) Infections (24.0%) Mechanical causes (19.7%) Others (22.1%)	17.8% (2.976/16.748) transfer to HD^c^ 8.4% (1.413/16.748) death^c^
Early technique failure in peritoneal dialysis patients in a multi‐ethnic Asian country (2020) International Urology and Nephrology. 52:1987–1994 https://doi.org/10.1007/s11255-020-02570-3 [17]	Peripheral vascular disease Etiology of ESKD	Death (41.8%) Infections (40.8%), Mechanical causes (10.2%) Other causes (7.1%)	10.8% (57/528) transfer to HD^c^ 7.8% (41/528) death^c^
*Factors associated with late pd discontinuation: other factors*			
Adapted from: time-dependent reasons for peritoneal dialysis technique failure and mortality (2010) Perit Dial Int.30:170–177 https://doi.org/10.3747/pdi.2008.00277 [8]	Loss of residual renal function Older age Diabetes, CV disease	Death (about 25%) Psychosocial/unknown (about 7–9%) Catheter complications (1–2%) Transplantation (42–43%) Infections (10–18%) Under dialysis/UFF/abdominal complications (4–7%)	24% (after 2 years)) 34% (after 3 years)
Predictors of outcomes in patients on peritoneal dialysis: a 2-year nationwide cohort study (2019) Sci. Rep. 9:3967 [14]	Older age Diabetes and CV comorbidities Higher C-reactive protein and phosphate levels Reduced serum albumin Use of 2.5% glucose dialysate	–	10% death 1.6% kidney transplantation 29.4% transfer to HD
Adapted from: peritoneal dialysis and mortality, kidney transplant, and transition to hemodialysis: trends from 1996–2015 in the United States (2020) Kidney Med. 2(5):610–619 https://doi.org/10.1016/j.xkme.2020.06.009 [12]	Small center (< 6 patients) Older age (> 65 years) African American Diabetes Congestive heart failure Cancer Tobacco use Alcohol/drug dependence	–	Discontinuation at 2 years: from 46% (1996 cohort) to 34% (2011 cohort) Unadjusted rates of transition from PD to CHD: from 24.5 in 1996 to 20.2 in 2013 (18% decrease); than increasing to 21.3 in 2014
Treatment practices and outcomes in incident peritoneal dialysis patients: the Swedish Renal Registry 2006–2015 (2021) Clinical Kidney Journal. 14(12):2539 2547 https://doi.org/10.1093/ckj/sfab130 [16]	–	–	10–17% transfer to HD^c^ 7.0–9.3% death^c^ 21–38% transfer to HD^e^ 15.8–18.7% death^e^
Predictors of technique failure and mortality on peritoneal dialysis: an analysis of New Zealand peritoneal dialysis registry data (2021) Nephrology. 26:530–540 https://doi.org/10.1111/nep.13837 [24]	Coiled tip catheters Small centers Late referral Creatinine clearance > 50 L/week	PD catheter-related infections (58.3%—of which 30% peritonitis) Surgical complications (10.8%) Inadequate dialysis (10.3%) Flow dysfunction (5.5%) Psychosocial factors (4.5%)	46.9% (2993/6379) transfer to HD
Analysis of risk factors and construction of prediction model of drop out from peritoneal dialysis (2021) Medicine. 100:3(e24195) https://doi.org/10.1097/MD.0000000000024195 [11]	Higher education level Diabetes Hypertension Repeated peritonitis Repeated chest complications	–	17.2% (65/377) transfer to HD 11.7% (44/377) kidney transplantation 10.3% (39/377) death
Peritoneal dialysis modality failure in a middle-income country: a retrospective cohort study (2021) Kidney Med. 3(3):335–342 https://doi.org/10.1016/j.xkme.2020.12.010 [10]	Diabetes History of major abdominal surgery Catheter implant technique Obesity Peritonitis	Catheter-related problems (27.3%) Peritonitis (25.4%) Psychosocial factors (20.6%)	6.9% transfer to HD^c^ 13.5% transfer to HD^e^ 19.6% transfer to HD^f^ Overall 22.6% (1462/6452)
Technique failure in peritoneal dialysis: Modifiable causes and patient-specific risk factors (2022) Perit Dial Int https://doi.org/10.1177/08968608221077461 [26]	Older age Higher comorbidity scores Ischemic heart disease CAPD	Leakage and catheter problems (15%^b^–5% later) Death PD-related infections (20%) Clearance problems (11–12%)	318/695 (of which 99 early discontinuation and 219 late discontinuation)
Importance of non-medical reasons for dropout in patients on peritoneal dialysis (2020) Clinical and Experimental Nephrology. 24:1050–1057 [27]	Age Diabetes PD performance by caregivers	Lack of caregivers Losing confidence in PD PD-related peritonitis	–

^a^After 3 months from PD start; ^b^after 6 months from PD start; ^c^after 12 months from PD start; ^e^after 24 months from PD start; ^f^after 36 months from PD start

In the last years it has been observed that even symptoms reported by patients could have clinical relevance and may be useful for improving patient care [21]. In this context, the Patient Reported Outcome Measures (PROMs) have shown some promise in predicting dialysis discontinuation [22]. A recent Chinese study [23] sought to investigate whether pre-dialysis symptoms are linked to mortality in patients on PD treatment. The authors used data of 898 patients (excluding patients who received HD for more than one month before PD start and those who had a kidney transplantation), and they observed that nausea was associated with all-cause mortality after 6 and 12 months from PD start while anorexia was the only symptom significantly associated with long-term mortality, after adjusting for confounding factors. These findings suggest that patients with nausea at dialysis start may benefit from more intensive treatment to prevent early discontinuation.

In summary, it is currently unclear what the pre-dialysis risk factors for PD discontinuation are. Available studies suggest that poorly controlled laboratory parameters such as serum calcium and albumin as well as clinical characteristics (obesity, older age) or PROMs may have an influence on PD outcomes. However, future endeavors should investigate whether these factors are causally related to PD discontinuation or, rather, represent markers of poor treatment adherence or compliance.

### Factors associated with early PD discontinuation

During the past 20 years, many studies have investigated the causes of early PD discontinuation (Table 2). As mentioned above, there is no globally accepted definition of early discontinuation, although a growing body of evidence defines early discontinuation as discontinuation which occurs within the first 6 months after PD start, according to the findings of Guo and Mujais previously described [19].

The Netherlands Cooperative Study on the Adequacy of Dialysis aimed at identifying demographic, clinical and laboratory factors, including data registered one month before dialysis start, linked to PD drop-out in different time periods [8]. A total of 585 patients who started PD and 124 patients who started HD but switched to PD within the first three months after RRT beginning between 1997 and 2007 were included in the study and stratified into four groups according to length of the follow-up: from 0 to 3 months (P1), from 4 to 12 months (P2), from 13 to 24 months (P3) and from 25 to 36 months of follow-up (P4). The primary end point of the study was PD discontinuation, defined as a permanent switch to HD or all-cause death on PD. The authors reported that in the first year of PD treatment the overall technique survival was 87%. In particular, the incidence rate of PD discontinuation was highest during the first three months and then remained stable for the next three years (Fig. 1). Switch to HD was the main cause of drop-out from PD in the first year, but from the third to the twelfth month there was a positive increase in transplantation rate (13% and 30% of the patients received a kidney transplant after 3 and 12 months following PD start, respectively). Transfer to HD decreased during the follow-up and it seemed that elderly and female patients with numerous comorbidities were more likely to switch to HD within the first three months of PD. The rate of catheter and abdominal complications (from 15 to 2% and from 7 to 2% for catheter and abdominal complications, respectively) as well as the drop-out rate for psychosocial/unknown reasons (from 20 to 7%) decreased from the first quarter on. Infections also decreased after the first year of treatment. Of note, the causes of death such as cardiac, vascular (including stroke and hemorrhage), infectious complications and abdominal complications not related to the treatment, PD-related complications and other reasons (including unknown causes, malignancy, and treatment refusal) did not differ among the four follow-up periods.

The French Language Peritoneal Dialysis Registry (RDPLF) investigators [9] retrospectively analyzed data of 9675 patients receiving dialysis between 2002 and 2010 in 138 French centers to investigate the causes of early PD discontinuation, defined as a transition to HD for more than 2 months in the first six months of PD. They considered death and transplantation as competing events. In this study, the authors observed that within 6 months of PD start, 6.3% of the patients had switched to HD, mostly due to catheter dysfunction (18.1%) and psychosocial reasons (16.4%). Other reasons were miscellaneous reasons related to PD (15.5%), peritonitis (15.3%), miscellaneous reasons unrelated to PD (13.3%), dialysis inadequacy (11.9%), ultrafiltration failure (7.8%) and malnutrition (1.8%). In the same time frame, 2.2% and 8.4% of patients were transplanted or died, respectively. Of note, while age, modified CCI and diabetes were associated with an increased risk of death and a lower probability of receiving a kidney transplantation, no association with early switch to HD (in the first six months from PD start) was detected. The authors also found that the PD technique (continuous ambulatory peritoneal dialysis vs automated peritoneal dialysis) was not associated with the likelihood of being transferred to HD in the first 6 months from PD inception.

Patients assisted by a caregiver had a higher risk of death and a lower probability of receiving a kidney transplantation, but they had the same risk of being switched to HD as other patients. Notably, RRT prior to PD was associated with technique survival depending on the type of RRT. Subjects who had been treated with HD before PD had a higher risk of being re-transferred to HD and to die than subjects treated with PD alone. However, patients resuming dialysis after failure of a kidney transplant had a higher probability of being re-transplanted and a lower risk of death, but they had a higher risk of PD discontinuation when compared to subjects with no history of kidney transplantation. Finally, early peritonitis (peritonitis within the first 6 months) and center size were linked to the risk of transfer to HD (lower risk being associated with larger centers, defined as centers with more than 20 new PD patients/year).

Another large study, which investigated the risk factors for early PD discontinuation, was conducted by the ANZDATA study group from Australia and New Zealand [13]. The authors included in the study 16,748 adult patients who initiated PD between 2000 and 2014 and observed them for one year. They divided the period of recruitment into three periods according to when subjects received dialysis: between the years 2000 and 2004, 2005 and 2009, and 2010 and 2014. The primary outcome was discontinuation within the first year of PD treatment defined as switch to HD for more than 30 days or death on PD or transfer to HD within 30 days from PD initiation. As secondary outcomes they investigated causes of discontinuation (death, infections, mechanical causes, other) within the first year of treatment. The authors found that 17.8% of patients switched to HD within the first year after PD start and 8.4% died, accounting globally for 26.2% of early discontinuation. Discontinuation was associated with age older than 70 years, White race, BMI < 18.5 kg/m^2^, history of ischemic heart disease, cerebrovascular disease or peripheral vascular disease, late referral to nephrology care (< 3 months before PD start) and center size (< 5 PD incident patients/year). There was no difference in the estimated glomerular filtration rate at the beginning of PD between patients who experienced discontinuation and those who did not. Of note, initiating PD between the years 2010 through 2014 was associated with lower risk of discontinuation when compared to beginning PD between the years 2000 through 2004.

Discontinuation due to mechanical complications are more frequent within the first 9 months and among patients with polycystic kidney disease, as also reported by the NECOSAD study investigators [8]. In the ANZDATA study, dialysate leak, development of hernias, and catheter dysfunction were commonly encountered during the first months of PD. However, though these complications may lead to PD discontinuation, the main causes of technique failure in the study were death by any cause and infections [8].

In another study, Sukul et al. analyzed data of incident PD patients registered from 1996 to 2015 in the US Renal Data System (USRDS) registry to define the rate of switch to HD (for at least 30 days), mortality, and transplantation [12]. For these analyses, about 200,000 subjects followed-up for three years were considered. To evaluate the historical trends, the study cohort was divided into 4 multiyear cohorts. Of note, the authors reported that in the 2 earliest cohorts, the rates of transition to HD were highest within the first 3 months and declined afterwards, while in the 2 more recent cohorts, the rates of transition to HD were slightly higher between the third and the sixth month after PD initiation. Similarly, a marked decline in mortality in more recent cohorts was also noted suggesting an overall improvement in the PD technique and/or teaching over time. Alternatively, the authors postulated that a selection of healthier subjects over time might also be responsible for these findings.

In 2020, a group of investigators from Singapore [17] analyzed the causes associated with early peritoneal dialysis discontinuation, defined as transfer to HD for at least 30 days or death, within the first year after PD start. The secondary outcomes were cause-specific early discontinuation, and death-censored discontinuation. They included 517 incident PD patients from 2013 to 2017 and followed them until 2018. In this study, 19.0% of patients had early discontinuation and the most frequent causes were death (41.8%) and infections (40.8%). They noticed, however, that individuals with peripheral vascular disease compared to those without it had a higher risk of early discontinuation. Unlike other reports, in this study, age, gender, diabetes, and ischemic heart disease were not associated with overall early discontinuation. However, male gender was significantly associated with a higher risk of PD-related infections, which was responsible for about 7.7% of the reported episodes of discontinuation. Similarly, all-cause mortality during the first year of PD, which was responsible for about 7.9% of PD dropouts, was significantly associated with peripheral vascular disease. Of note, patients with ESKD due to glomerulonephritis had a lower risk of discontinuation caused by death compared with ESKD due to hypertension.

Whether advancements of PD techniques over the years is associated with a parallel improvement in patient outcomes was investigated by Xu et al. [16] who analyzed data of 3122 patients undergoing peritoneal dialysis between 2006 and 2015 in Sweden. Investigators divided the length of observation into five 2-year periods. The primary outcome was one- and two-year all-cause mortality and major adverse cardiovascular events ([MACEs]—composite of cardiovascular mortality, myocardial infarction, hospitalization for heart failure and stroke), while the secondary outcomes were rate of kidney transplantation, switch to HD, and PD-related peritonitis occurring one and two years after PD inception. They found that the incidence rates of death (about 8%) and MACEs (about 13%) as well as the proportion of patients transferred to HD after one year (about 13%) were stable during the 10-year index period. Notably, in the same period the incidence of peritonitis during the first-year of PD treatment decreased over time (21% in the years 2006–2007 vs 18% in the years 2014–2015). In contrast, the number of kidney transplants increased (6.8% for the years 2006–2007 vs 10.3% for the years 2014–2015) in the same time frame. The same results were found by analyzing data two years after starting PD.

Although these studies used different definitions of PD discontinuation and analyzed different outcomes, the most common risk factors related to early PD discontinuation are older age, presence of several comorbidities, center size (less than 10–20 new PD patients/year) and late referral to nephrology care. On the other hand, mechanical complications and early peritonitis are the most common reasons for RRT modality change (switch to HD).

### Factors associated with late PD discontinuation: other factors

As “other factors”, we consider factors which lead to PD discontinuation regardless of the period after PD start and/or after the first year from PD start (Table 2).

The NECOSAD study group [8] analyzed different periods of PD treatment (*see above for a detailed description*) and they found that technique survival was 87%, 76% and 66% one, two and 3 years after PD start. Death contributed to discontinuation for about 25–30% in all the considered periods. After the first year of dialysis, the percentages of patients who underwent a kidney transplant increased and were 42% and 43% in the 2 and 3 years following PD start. Unlike technique complications, underdialysis and ultrafiltration failure increased with time on PD. Of note, residual renal function had a significant influence on technique survival during the second year of PD treatment.

A study conducted in Japan using data from the registry of the Japanese Society for Dialysis Therapy (JSDT), analyzed the causes of death of 8,945 prevalent PD patients receiving RRT between 2014 and 2015 [14]. During the 2-year study period, only 1.6% of the study cohort received a kidney transplant, while about 10.0% and 29.4% of study subjects died or were transferred to hemodialysis, respectively. Notably, only 58.9% were alive at the end of the study period. At multivariable adjusted analyses, older age, longer duration of PD, presence of diabetes, cardiovascular comorbidities, the use of 2.5% glucose dialysate, higher C-reactive protein and phosphate levels as well as lower serum albumin were factors independently associated with a higher risk of all-cause death. Of interest, diabetes mellitus was associated with a 50% increased risk of death, irrespective of adjustment for demographic, clinical, dialysis adequacy and nutritional factors. In the index period, 1,240 episodes of PD-related peritonitis occurred in 859 patients (mean peritonitis rate was 0.18 per patient-year). Previous history of peritonitis, use of a connecting device, comorbid cadiovascular disease, and higher BMI were independent predictors of peritonitis.

In the USRDS registry, [12] an increase in the median technique survival from the 1996 to 2011 cohort was observed. In particular, the unadjusted rates of transition to HD decreased from 24.5% in 1996 to 21.3% in 2014. The identified factors associated with transition to HD were older age, African American ethnicity, presence of diabetes, congestive heart failure, cancer, tobacco use, and alcohol/drug dependence. Death rate also declined from 20.2% in 1996 to 11.0% in 2008, with a smaller decrease in the years considered afterward. While male sex and non-White ethnicity had the lowest risk of death, mortality rate increased with longer time on PD. Similarly to what was previously reported, centers with a lower number of patients on PD (i.e., centers treating 6 or fewer patients compared with centers treating 25 or more patients) had higher rates of switch to HD and death. Interestingly, transplantation rate declined from 10.4 in 1996 to 6.8 in 2014, and it was less likely to occur in older, non-White, female, and Hispanic patients.

In another report from the New Zealand Peritoneal dialysis registry (NZPDR) [24], 3,165 and 3,214 patients who initiated PD in two historical periods (between 1995 to 2004 and between 2005 to 2014) were analyzed to investigate time and causes of discontinuation (intended as transfer to HD for at least 90 days) as well as time to death. Overall, 2,993 episodes of discontinuation mainly due to infections (58.3%), followed by catheter dysfunction, inadequate dialysis, surgical complications, and psychosocial factors were registered. Of note, the number of peritonitis cases was significantly lower in the second period compared to the first one. Factors associated with a lower risk of PD discontinuation were age over 60 years, Asian ethnicity, large center size as well as the presence of renovascular disease, diabetes mellitus and continuous ambulatory peritoneal dialysis (CAPD). The number of deaths was 2684 and older age, renovascular disease and diabetes were associated with higher mortality rates. In contrast, female gender, Asian and Pacific ethnicity as well as CAPD were associated with lower risk of death. The risk of compound outcomes (death and PD discontinuation) was higher in patients over 60 years of age and in those with diabetes.

A group of investigators from Colombia investigated the risk factors associated with discontinuation and its frequency [10] in 6452 adult patients who started PD between 2010 and 2015 and were followed-up until December 2018 at the Renal Therapy Services network in Colombia. Discontinuation was defined as a switch to hemodialysis for at least 30 days. Death and transplantation were analyzed as competing-risks events. During follow-up, 1,462 events of discontinuation (10.6 events/100 patients-years) occurred. Notably, a stepwise increase in the incidence of PD discontinuation (adjusting for competing risks) during the first 3 years of PD was noted (6.9%, 13.5% and 19.5% at the end of the first, second and third year of PD, respectively). Moreover, these events were more frequent during the first 40 days after PD start, and the most common causes of discontinuation were catheter complications (catheter obstruction due to fibrin, omentum, adhesions, as well as catheter displacement), peritonitis, and psychosocial/medical indications (especially in the first 90 days). In the multivariable analysis, diabetes, history of major abdominal surgery, catheter implant technique, obesity and peritonitis were all related to higher risk of discontinuation. In particular, patients who had at least one episode of peritonitis during the follow-up had a twofold increased risk of discontinuation when compared to patients who did not experience peritonitis.

In Italy, a national census is conducted every 2 years by the Peritoneal Dialysis Study Group to analyze the situation of PD in the country. In the census published in 2019 [25], relative to the year 2016 and considering data from 237 public hospitals, the authors noticed that the number of PD dropouts, both overall and for individual causes, had not changed significantly over the years. In particular, 521 deaths (11.8 ep/pt-yrs), 554 transfers to HD (12.5 ep/pt-yrs) and 311 kidney transplants (7.0 ep/pt-yrs) were registered. The most frequent cause of dropout and switch to HD was peritonitis, although the percentage is significantly reduced compared to the previous years. In this census it was noticed that the dropout due to choice or inability to continue was an important cause of PD discontinuation. Moreover, the percentage of dropout due to insufficient ultrafiltration and catheter malfunction remained stable, while the prevalence of insufficient purification increased from 2005 to 2016.

In a Chinese retrospective study conducted by Li et al. including 377 patients who underwent peritoneal catheter implantation and peritoneal dialysis between 2009 and 2019 [11], the most common reason for discontinuation was switch to hemodialysis (41.67%), followed by kidney transplant (28.21%) and death (25.00%). At univariate analysis, age, ethnicity, marital status, obstructive nephropathy as cause of ESKD, and urinary tract infection during peritoneal dialysis were not associated with dropout from PD, whereas female gender, medium or high education level, diabetes, glomerulonephritis and hypertension as causes of ESKD, low number of hospitalizations after catheterization, peritonitis and repeated chest complications (pulmonary infection and pleural effusion due to thoracic and abdominal leaks) were risk factors for cessation of peritoneal dialysis. At multivariate analysis, all factors with the exception of female gender remained independently associated with PD dropout. Of interest, the authors created a nomogram to predict the prognosis of patients receiving PD. Indeed, level of education combined with history of diabetes, hypertension, peritonitis, chest complications, and number of hospitalizations after catheterization predicted PD discontinuation with high reliability (C-index of 0.74). However, the lack of external validation of this scoring system limits its applicability in other PD populations or in daily practice.

The DOMESTICO (Dutch nOcturnal and hoME dialysis Study To Improve Clinical Outcomes) study [26] published in 2022 included 695 patients from 22 different centers who started and continued PD for a minimum of 14 days between 2012 and 2016, and followed them up until 1 January, 2017. The authors defined a composite of transfer to in-center HD (CHD) for more than 30 days, death on PD or death within 30 days after transfer to HD as PD discontinuation (primary endpoint of the study), while the secondary endpoints of the study were defined as death-censored discontinuation, all-cause of death, and permanent discontinuation (composite of transfer to CHD for at least 180 days, death on PD or death within 180 days after transfer to CHD). Patients were stratified as early discontinuation (defined as discontinuation occurring within the first 6 months on PD) and late discontinuation (discontinuation more than 6 months after PD start). At study completion, 318/695 patients had experienced discontinuation. In particular, 99 patients developed early discontinuation while 219 patients developed late discontinuation. The rate of discontinuation during the first year was 29% which increased to 52% at the end of the second year of PD treatment. Factors associated with discontinuation were older age, presence of comorbid conditions such as ischemic heart disease, and CAPD. The most frequent cause of discontinuation was death followed by PD-related infections (peritonitis, exit-site infections). Notably, catheter-related problems accounted for a large number (15%) of PD discontinuation episodes during the first six months of PD while the occurrence of these complications was significantly reduced (5%) in the following months of PD. A similar trend was noted for fluid leakage, whereas infections and dialysis adequacy were important causes of both early and late PD discontinuation. Death censored discontinuation increased over time on dialysis (23% and 35% during the first and second year, respectively) and the most common causes of death were cardiovascular diseases (28%), non PD-related infections (15%), and malignancies (13%) Finally, permanent PD discontinuation occurred in 254 patients predominantly due to death and infections by study completion.

The impact of non-medical reasons (i.e., lack of a caregiver and/or losing confidence in PD) on PD discontinuation (defined as switch to HD or death) was tested in a registry study conducted in Taiwan between 2014 and 2018 [27]. A total of 224 patients receiving PD at the Kaohsiung Chang Gung Memorial Hospital in Taiwan were identified. Of these, 187 and 37 dropped out for medical and non-medical reasons, respectively. The two leading non-medical reasons were lack of caregivers (*n* = 12) and losing confidence in PD (*n* = 10). Other non-medical reasons included burnout (*n* = 4), not qualified PD manipulation by PD training nurses (*n* = 4), concern about overall appearance (*n* = 3), transfer to nursing home on family request (*n* = 2), transfer to HD by patient request (*n* = 1), and discomfort at social events (*n* = 1). In contrast, PD-related peritonitis (*n* = 101) was the main medical reason for PD dropout. Of interest, the unadjusted cumulative dropout rate from PD was higher for non-medical vs medical reasons for stopping PD, although patients terminating PD for non-medical-reasons were older, more likely to be diabetic and having been dialyzed for a shorter period of time. While these results warrant confirmation, they suggest that also logistic or non-medical factors may play a role in PD retention.

In summary, interpretation of available data is complicated by the use of different statistical methods and definitions of PD discontinuation. Indeed, some studies used a competitive risk approach while others included death and/or transplantation in the definition of PD discontinuation as these outcomes would have the same clinical impact at the patient level. Nevertheless, overall factors associated with late switch to HD or death include older age, higher disease burden and small center size. Unlike what has been reported for early PD discontinuation, the willingness of the patients or of the caregiver to continue PD, as well as the number of hospitalizations are relevant determinants for patient burnout and late PD discontinuation.

## Conclusions and future perspectives

A growing body of evidence indicates that patients on PD have comparable (or better) clinical outcomes and quality of life as patients on HD [2, 4, 5, 7, 28]. In addition, PD is generally less expensive than HD, making this RRT a cost-effective option for ESKD patients. However, several medical as well as personal or non-medical factors (Fig. 1) may limit its implementation, and currently less than 20% of patients with ESKD benefit from this treatment. In this regard, in-depth knowledge of factors that might influence the outcome of patients on PD may ease access to PD and be of use to advise ESKD patients facing RRT. In addition, it may help in the timely management of potential complications that could eventually result in PD discontinuation or patient burnout.

However, our review highlights several limitations for the implementation of an evidence-based approach towards the selection of the most appropriate dialysis modality. Indeed, the lack of a standardized definition of PD discontinuation complicates the comparison of different study results as well as the understanding of the clinical relevance of various parameters or factors associated with PD outcome. Nevertheless, by comparing studies performed in different settings over the last decade, we consistently identified some risk factors associated with PD discontinuation (Fig. 1) that may affect PD outcome. Although PD-related infections (i.e., peritonitis and exit site infections) are a major cause of PD dropout regardless of the time spent on dialysis, catheter and mechanical complications are the main causes of PD termination in the early phase of treatment (first 6 months of treatment). Besides the patient comorbidity burden, in this phase, patient training in a large PD center (with more than 10–20 patients) as well as the PD modality may modulate the risk of interrupting PD treatment. In a later phase (after 6 months of PD), death, switch to HD due to underdialysis and ultrafiltration failure, but also transplantation represent the major reasons for abandoning the PD program. Psychosocial factors (i.e., patient burnout) as well as the need for a caregiver, or frequent hospitalizations may be independent factors for PD termination, especially in elderly and frail, comorbid patients (Fig. 1). Of note, no study has investigated the impact of the time spent on PD on clinically relevant outcome and whether HD switch should be considered after a certain number of months on PD. Rather, it seems that the decision of RRT technique change should be made in case of PD failure and that it should be addressed on a single case-level. Similarly, only few studies have investigated the impact of the transition from PD to home HD vs in-center HD, and future endeavors are thus needed to shed light on the best course of action when PD cannot be continued[29, 31].

To improve PD utilization, future studies adopting common evidence-based definitions of outcome are required to better elucidate modifiable and unmodifiable risk factors in different phases of PD. In this regard, whether home-to-home RRT transition can favor PD implementation and improve the patient’s quality and quantity of life also needs to be addressed, as suggested by two observational studies [29–31].

In summary, to prevent PD discontinuation, physicians should closely monitor older, frail and comorbid patients, offer periodic refresher courses on PD technique to reduce the risk of infections, as well as provide psychological support to patients and caregivers to prevent burnout, especially in patients undergoing numerous hospitalizations. Finally, a multifaceted approach involving the joint efforts of governments, patients and physicians and other stakeholders is desirable to overcome the psychosocial and logistic barriers that may result in discontinuation of the dialysis program.

## Abbreviations

APD: Automated peritoneal dialysis
CAPD: Continuous ambulatory peritoneal dialysis
CCI: Charlson comorbidity index
CHD: In-center HD
DOMESTICO: Dutch nOcturnal and hoME dialysis Study To Improve Clinical Outcomes
ESKD: End stage kidney disease
HD: Hemodialysis
JSDT: Japanese Society for Dialysis Therapy
MACEs: Composite endpoint of cardiovascular mortality, myocardial infarction, hospitalization for heart failure and stroke
NECOSAD: The Netherlands cooperative study on the adequacy of dialysis study
NZPDR: New Zealand Peritoneal Dialysis Registry
PD: Peritoneal dialysis
PROMs: Patient reported outcome measures
RRT: Renal replacement therapy
UFF: Ultrafiltration failure
